# The Influence of Clinical Information in the Histopathologic Diagnosis of Melanocytic Skin Neoplasms

**DOI:** 10.1371/journal.pone.0005375

**Published:** 2009-04-30

**Authors:** Gerardo Ferrara, Zsolt Argenyi, Giuseppe Argenziano, Rino Cerio, Lorenzo Cerroni, Arturo Di Blasi, Elisa A. A. Feudale, Caterina M. Giorgio, Cesare Massone, Oscar Nappi, Carlo Tomasini, Carmelo Urso, Iris Zalaudek, Harald Kittler, H. Peter Soyer

**Affiliations:** 1 Department of Pathology, Gaetano Rummo General Hospital, Benevento, Italy; 2 Department of Dermatology, University of Washington, Seattle, Washington, United States of America; 3 Department of Dermatology, Second University of Naples, Naples, Italy; 4 Department of Dermatology, University of London, London, United Kingdom; 5 Department of Dermatology, Medical University of Graz, Graz, Austria; 6 Department of Pathology, Basilicata Oncology Reference Centre, Rionero in Vulture, Italy; 7 Department of Pathology, Antonio Cardarelli General Hospital, Naples, Italy; 8 Department of Biomedical Sciences and Human Oncology, Second Dermatologic Division, University of Turin, Turin, Italy; 9 Department of Pathology, Dermatopathology Section, S.M. Annunziata Hospital, Florence, Italy; 10 Department of Dermatology, Division of General Dermatology, Medical University of Vienna, Vienna, Austria; 11 Dermatology Research Centre, The University of Queensland, School of Medicine, Princess Alexandra Hospital, Brisbane, Australia; Uppsala University, Sweden

## Abstract

**Background:**

We tested the relevance of clinical information in the histopathologic evaluation of melanocytic skin neoplasm (MSN).

**Methods:**

Histopathologic specimens from 99 clinically atypical MSN were circulated among ten histopathologists; each case had clinical information available in a database with a five-step procedure (no information; age/sex/location; clinical diagnosis; clinical image; dermoscopic image); each step had a histopathologic diagnosis (D1 through D5); each diagnostic step had a level of diagnostic confidence (LDC) ranging from 1 (no diagnostic certainty) to 5 (absolute diagnostic certainty). The comparison of the LDC was employed with an analysis of variance (ANOVA) for repeated measures.

**Findings:**

In D1 (no information), 36/99 cases (36.3%) had unanimous diagnosis; in D5 (full information available), 51/99 cases (51.5%) had unanimous diagnosis (*p* for difference between proportions <0.001). The observer agreement expressed as kappa increased significantly from D1 to D5. The mean LDC linearly increased for each observer from D1 through D5 (*p* for linear trend <0.001). On average, each histopathologist changed his initial diagnosis in 7 cases (range: 2–23). Most diagnostic changes were in D2 (age/sex/location).

**Interpretation:**

The histopathologic criteria for the diagnosis of MSN can work as such, but the final histopathologic diagnosis is a clinically-aided interpretation. Clinical data sometimes reverse the initial histopathologic evaluation.

## Introduction

The histopathologic diagnosis of melanocytic skin neoplasms (MSN) is often matter of considerable debate, even among experienced histopathologists [1]–[3]. As a general rule, no clinical information should ever reverse a histopathologic diagnosis when the microscopic features are clear-cut. Nonetheless, basic clinical information about any MSN (age and sex of patient; location of the lesion) are required and commonly used [4] by histopathologists in their routine practice, particularly when approaching peculiar MSN, such as early biopsied congenital naevi [5], [6] and spitzoid lesions [4], [7]. Moreover, the existence of the so-called ‘special sites’ of MSN [8] clearly means that the location of the lesions is another important diagnostic criterion. In recent years, following the increasing use of dermoscopy (dermatoscopy, epiluminescence microscopy, skin surface microscopy) for the preoperative evaluation of MSN [9]–[14], several reports have shown the positive influence of the dermoscopic features on the histopathologic evaluation of MSN [15]–[19].

These results emphasize the value in studying whether clinical history and clinical information would impact upon the histopathologic diagnosis in dermatopathology [20].

We herein present the first study aimed at formally evaluating the influence of clinical data in routine histopathologic evaluation of MSN.

## Methods

### Study design

The goal of the present study was the evaluation of the importance of the clinicopathologic correlation in the diagnosis of MSN. A series of MSN was selected on the basis of clinical criteria. A panel of histopathologists received the respective microscopic slides together with a database which gave them a sequential access to clinical information for each case.

### Clinical and histopathologic material

Two of us (GA and IZ) retrieved cases of skin neoplasms consecutively excised from January 2004 to December 2005 for routine histopathologic examination. Exclusion criteria were: a) melanocytic naevi excised for cosmetic reasons; b) non-melanocytic lesions, as documented by the original histopathologic report. Thus, only clinically and/or dermoscopically atypical MSN entered the study. Each retrieved case had complete clinical information, comprising digitized clinical and dermoscopic images. The latter were JPEG-compressed files, 2048×1360 pixels in size, 300 dpi in resolution, obtained using a digital camera (Nikon Coolpix 995) coupled with Dermlite Foto lens (3-Gen, Salvador Bay, Dana Point, CA, USA) for dermoscopic imaging.

One of the authors (GF) provided the original histologic material of the selected cases and discussed them with a histopathologist expert in dermoscopy (CM) in order to choose for each case, a single paraffin block as being representative of the given lesion. New haematoxylin-eosin stained microscopic slides were prepared from the chosen blocks and again checked for their technical as well as for their diagnostic adequacy.

Submission of clinical and histopathologic materials to further diagnostic consultations was authorized by the patients or their guardians.

### Database preparation and functions

All the clinical information concerning the selected cases was included into a FileMaker Pro 7™ (FileMaker Inc.) generated database. For each case a sequential access to the clinical information was employed according to a five-step procedure, with a diagnosis (D) given for each step:

***D1:*** Diagnosis with no information available.
***D2:*** Diagnosis with knowledge of age and sex of patient, as well as location of the lesion.
***D3:*** Diagnosis with knowledge of the clinical diagnosis, as made in agreement by two of us (GA and IZ).
***D4:*** Diagnosis with the clinical image available.
***D5:*** Diagnosis with the dermoscopic image available

Case by case, from D1 through D5 the histopathologists were asked to log into the database also a *level of diagnostic confidence* (LDC), namely, the probability, as scored according to an arbitrary scale, that they ‘subjectively’ attributed to the given diagnosis [21]. The LDC scale was structured into five levels:

***LDC 1*** – No diagnostic certainty: no diagnosis can be made.
***LDC 2*** – Low diagnostic certainty: a diagnosis is felt as slightly more likely.
***LDC 3*** – Moderate diagnostic certainty: a diagnosis is favoured, but with some elements of doubt.
***LDC 4*** – High diagnostic certainty: a diagnosis is strongly favoured.
***LDC 5*** – Absolute diagnostic certainty: no other diagnosis is possible.

Therefore, each case had five diagnoses (D1 through D5) and each diagnosis had its LDC (ranging from 1 to 5).

### Histopathology panelists

Ten histopathologists were invited to study the cases: among these, five histopathologists had no specific experience in clinical dermatology (ADB, EAAF, ON, CT, CU), whereas the other five histopathologists did have clinical expertise (ZA, RC, LC, HK, HPS). Two histopathologists with no clinical experience (ADB and ON) and two histopathologists with clinical expertise (LC and HPS) had previously worked at the same Institution for several years; the other panelists had been never working in cooperation.

### Statistical Analysis

For statistical analysis, all the diagnoses were grouped into three ratings: ‘melanoma’, ‘naevus’, and ‘unknown’. Consensus diagnosis was defined as a diagnosis made in agreement by at least seven out of ten panelists, regardless the reported LDC.

Given *n* = 10 as the number of panelists, the interobserver agreement among the *n*(*n*−1)/2 = 45 possible pairs of observers was evaluated by using the *k* statistics introduced by Cohen [22]. The *k* statistics for multiple observers and the standard errors for were calculated by using the method reported by Fleiss [23]–[25]: *k* values range between +1 (perfect agreement) and −1 (perfect disagreement); values greater than 0.75 represent an excellent agreement; values lower than 0.40 a poor agreement; and values between 0.40 and 0.75 a fair to good agreement beyond chance. For the comparison of the LDC we used an analysis of variance (ANOVA) for repeated measures. All given *p*-values are 2-tailed and a *p*-value of <0.05 indicates statistical significance.

A change of diagnosis during the stepwise examination of a given case was defined as a switch from ‘naevus’ to ‘melanoma’ or vice versa, or else, as a change from ‘unknown’ to either ‘naevus’ or ‘melanoma’.

## Results

The study included 99 cases from 96 patients (M: F = 0.6:1; age range: 10–78 years; mean age: 43.3 years; median age: 42 years). Three patients had two lesions each which had been excised in different times; the histopathology panelists were unaware that these different lesions were from the same patients. The most common location was the back (40 cases), followed by the lower limbs (16 cases) and by the upper limbs (12 cases). The original histopathologic diagnoses rendered by one of the authors (GF) were ‘naevus’ in 54 cases and ‘melanoma’ in 45 cases.

Regardless the LDC, the study of the histopathologic specimens alone (D1) gave a consensus diagnosis in 89/99 cases (89.9%), with 55 cases diagnosed as naevus and 34 cases as melanoma. Complete agreement in D1 occurred in 36/99 cases (36.3%); of these, 17 were diagnosed as naevus and 19 as melanoma. The *k* value for the chance-corrected agreement in D1 was 0.57 (95% CI: 0.54–0.60).

**Figure 1:**
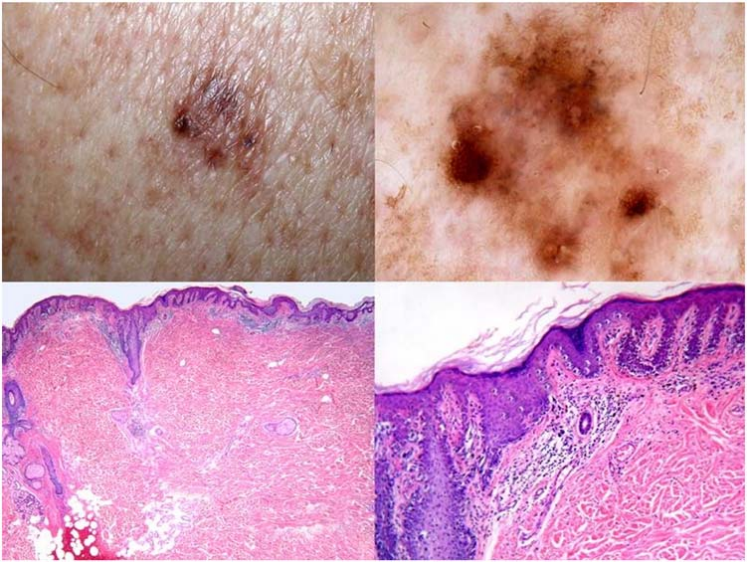
A 69-year-old man with a lesion from the back showing clinical (top left) and dermoscopic (top right) features of regression [16]. Histopathologically, the lesion is medium to large in size and shows a regular epidermal hyperplasia (bottom left). The main feature of atypia is the presence of areas of prevailing single cell proliferation at the junction (bottom right). Lentiginous melanocytic proliferations of the elderly are often controversial from both a both conceptual and a practical point of view. The lesion at issue was diagnosed as melanoma in situ, lentiginous type, [19] by six histopathologists in D1 and by eight histopathologists in D5.

**Figure 2:**
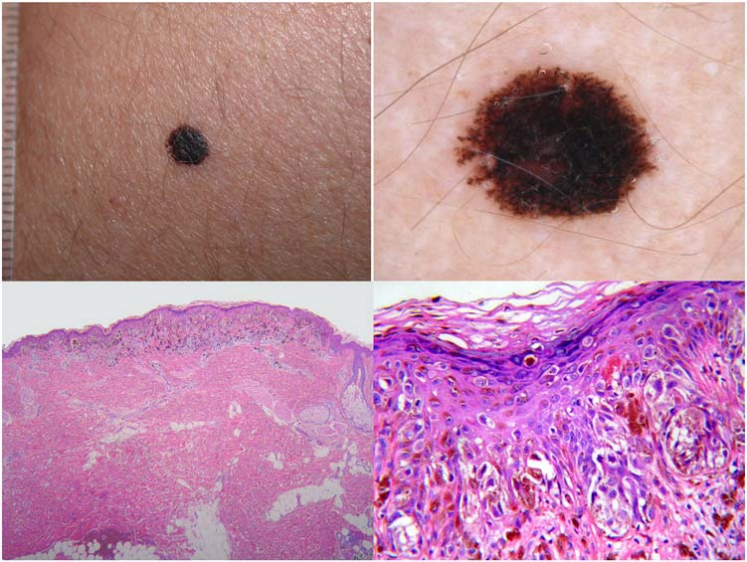
A 20-year-old woman with a lesion from the leg showing clinical (top left) and dermoscopic (top right) features consistent with pigmented Spitz naevus [18]. Histopathology revealed a sharply circumscribed, medium-sized lesion which was mainly characterized by nests of melanocytes at the junction (bottom left; haematoxylin-eosin, ×40). However, in some worrisome microscopic fields, melanocytes at all levels of the epidermis were seen (bottom right; haematoxylin-eosin, ×250). Due to these conflicting features, in the absence of any clinical information the lesion was diagnosed as melanoma by three histopathologists and as naevus by seven histopathologists. With the knowledge of the complete clinical information, the lesion was finally diagnosed as benign by all the histopathologists.

The number of the consensus diagnoses in D5 increased only slightly (91/99 cases [91.9%]) with 55 cases diagnosed as naevus and 36 cases diagnosed as melanoma. Therefore, compared with D1, two additional cases had a consensus diagnosis of melanoma in D5. Both cases were lentiginous melanocytic proliferations of the elderly. Figure 1 illustrates one of these cases. Complete agreement in D5 strikingly increased to 51/99 cases (51.5%), with 24 cases diagnosed as naevus and 27 cases diagnosed as melanoma (*p* for difference between proportions <0.001). The *k* value increased from D1 to D5 and reached 0.67 (95% CI: 0.64–0.70) in D5 which is significantly higher than in D1. The number of cases labeled as “unknown” by any of the observers decreased from 32 in D1 to one case in D5 (Table 1). Figure 2 shows a case in which a unanimous diagnosis was reached only at the end of the five-step procedure.

**Table 1:** Agreement (kappa) at every stage of diagnosis and number of “unknowns”.

	Overall Agreement (Kappa)	95% CI	Agreement for category nevus (kappa)	95% CI	Agreement for category melanoma (kappa)	95%CI	Category „Unknown” (n)
D1	0,57	0,54–0,60	0,58	0,43–0,74	0,63	0,51–0,76	32
D2	0,64	0,61–0,66	0,64	0,48–0,79	0,66	0,53–0,78	9
D3	0,65	0,62–0,67	0,64	0,49–0,80	0,67	0,54–0,79	7
D4	0,66	0,64–0,69	0,67	0,51–0,82	0,67	0,54–0,80	3
D5	0,67	0,64–0,70	0,67	0,51–0,83	0,67	0,54–0,80	1

**Figure 3:**
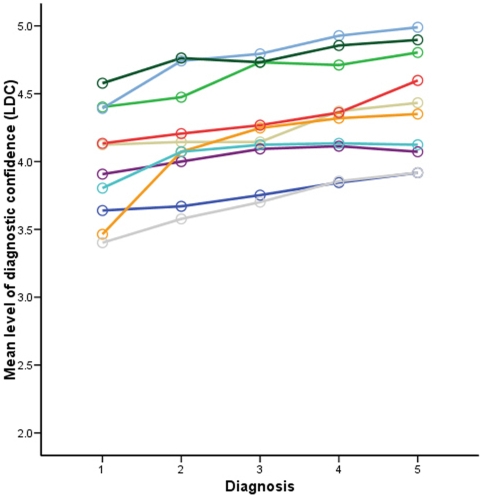
The increase of the mean LDC for each histopathologist according to the diagnostic steps. On the horizontal axis: 1 – No clinical information. 2 – Age and sex of the patient; location of the lesion. 3 – Clinical diagnosis. 4 – Clinical image. 5 – Dermoscopic image.

The mean LDC among all the panelists was 3.9 in D1 and 4.4 in D5. It increased in a linear fashion for each observer after each step of additional information given (Figure 3). The increase in the level of confidence was statistically significant for the whole group (*p* for linear trend <0.001).

**Figure 4:**
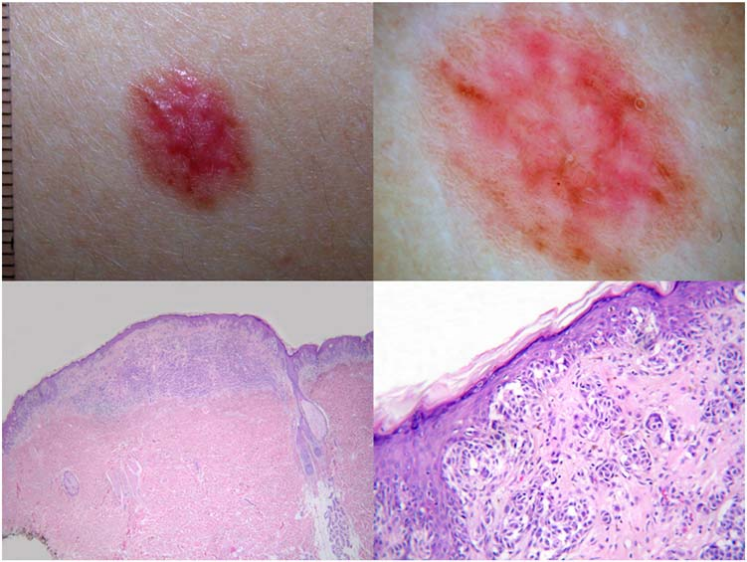
A hypopigmented lesion (top left) from the back in an 18-year-old woman dermoscopically showing atypical vessels and reticular depigmentation (top right). Histopathology reveals a large-sized and asymmetric lesion with focal flattening of the dermoepidermal junction (bottom left); in some areas there is some irregular spacing of junctional nests and some pagetoid spreading (bottom right). The lesion was finally diagnosed as congenital nevus by all the histopathologists; two of these panelists changed in D2 their first diagnosis of ‘unknown’.

The ten panelists gave a total of 990 diagnoses per step and 4950 diagnoses for the entire study. Table 2 itemizes the number of changes of diagnosis following each step of clinical information. Changes of diagnosis from D1 through D5 occurred in 87/4950 instances (1.75%). On average, each observer changed his initial diagnosis in 7 cases (range of changes: 2–23). Table 2 shows that most changes (49/87; 56.3%) occurred in D2 (knowledge of age and sex of the patient and location of the lesion), with a diagnostic switch into ‘naevus’ in 26 instances and into ‘melanoma’ in 22 instances. Overall, ten out of 99 cases received more than one change of diagnosis in D2. Five of these cases were lentiginous melanocytic proliferations (one of these cases is illustrated in Figure 1); three cases were lesions with Spitz/Reed nevus-like features (one of these cases is illustrated in Figure 2). The remaining two cases were congenital nevi with an atypical junctional component (one of these cases is illustrated in Figure 4).

**Table 2:** Change of diagnosis following provision of clinical information.

Diagnostic change		Diagnostic information				
		D2	D3	D4	D5	Total
*Into benign*	*Unknown to naevus*	14	1	3	2	20
	*Melanoma to naevus*	12	4	4	3	23
*Into malignant*	*Unknown to melanoma*	11	0	1	0	12
	*Naevus to melanoma*	11	3	11	6	31
*Into unknown*	*Nevus to unknown*	0	0	0	0	0
	*Melanoma to unknown*	1	0	0	0	1
	*Total*	49	8	19	11	87

Changes in D3 (clinical diagnosis), in D4 (clinical image), and in D5 (dermoscopic image) occurred 8 (9.1%), 19 (21.8%), and 11 (12.6%) times, respectively. After viewing both clinical and dermoscopic images, 12 diagnoses were switched into ‘naevus’ and 18 diagnoses were switched into ‘melanoma’.

## Discussion

The present study demonstrates that the histopathologic criteria in the diagnosis of MSN can work as such, but the final diagnosis is a clinically-aided interpretation. Clinical information can change the histopathologic diagnosis in a number of cases.

Whether any diagnostic test should be read together with clinical information has been debated since 1963, when Schreiber [26] suggested that clinical information improved the accuracy of chest X-ray evaluation. A commonly used argument against the ‘interdisciplinary’ approach is that clinical information may bias the reading [20], [27].

Very few studies have been carried out in cytohistopathology for the evaluation of the diagnostic impact of the clinical information [28], [29]. However, several criteria routinely adopted for the histopathologic diagnosis of MSN are implicitly based on clinicopathologic correlation. Early biopsied congenital naevi can show morphologic features – i.e.: proliferative nodules [5] and pagetoid extension [6] – which, in a completely different clinical context, would probably warrant a diagnosis of melanoma. A trend has been observed in interpreting almost all spitzoid lesions in children as ‘Spitz naevi’ and many spitzoid lesions in adults (particularly beyond the age of 30 to 40 years) as melanoma [4], [7]. There is a growing list of ‘special sites’ for naevi (acral skin, knees, genital area, mammary region, cutaneous folds, nail matrix, conjunctiva) [8]: MSN arising in these ‘special sites’ are characterized by histopathologic features which could become worrisome and favour the diagnosis of melanoma in ‘non-special sites’.

It has been recently claimed that dermoscopy is the conceptual and practical link between clinical dermatology (macrocosm) and dermatopathology (microcosm) [9], [14]. Like clinical dermatology, dermoscopy works in parallel to the skin surface; like histopathology, it allows to visualize structures which could not be discernible by the naked eye. Dermoscopy can therefore draw the attention of histopathologists to the suspicious areas of a given MSN [15] thus orienting the macroscopic sampling and/or suggesting the need of step-sectioning the paraffin block(s).

In the present study, most cases received a consensus diagnosis (at least 7/10 diagnoses in agreement) even in the absence of any clinical information. The chance-corrected interobserver agreement expressed as Fleiss' *k*appa was 0.57 even in this phase, and largely overlapping with the results of the previously reported studies on this topic [1]–[3]. The number of cases with a consensus diagnosis, as well as the *k* statistics significantly higher when all the clinical information were available, suggesting that the histopathologic diagnosis can be improved by the clinical data.

Clinical data were important in order to increase the LDC and this increase was statistically significant for all the observers regardless of their clinical expertise. Clinical information may influence microscopic observation at two stages: *perception* (identification of abnormal areas and their features) and *interpretation* (attribution of the abnormalities to an entity). Mainstream arguments state that clinical information, when evaluated before the microscopic study, can influence both phases, therefore biasing the microscopic study [20]. This study demonstrates that histopathologists can apply their criteria in the absence of any clinical information (see above), and can therefore have an *unbiased perception* of any given MSN. However, they feel more confident with their diagnosis by means of a *clinically-aided interpretation* [30].

Ideally, the clinical course should allow inferring the biologic potential of a given MSN. However, ‘malignancy’ not invariably implies ‘metastasis’ and death’; this is why clinicopathologic studies on MSN often refer to the histopathologic diagnosis as the ‘golden standard’ [13]. However, based on the evaluation of the histopathologic interobserver agreement, it was already demonstrated that there is the need for a better standardization and greater reproducibility in the histopathologic diagnosis of MSN [1]–[3]. Therefore, the histopathologic diagnosis is not a true ‘golden standard’ but rather an *‘assessment of probability’*: this means that we should refer to the histopathologic report to as a ‘diagnostic proposal’ within a decision-making process. The latter should incorporate, together with the ‘histopathologic diagnostic proposal’, all pertinent clinical data, both from the patient (personal history, age, sex, presence of clinically similar lesions) and from the lesion under examination (location, history of changes, clinical and dermoscopic features). We have adjusted the design of the study to deal with the situation of a lack of a gold standard (or practical reference standard). Since “correctness” is not available we have chosen to look for indirect measures of the quality of a diagnosis, namely, agreement of independent experts, and confidence in the diagnosis. To minimize bias we have chosen independent experts, from different schools, who are not working together. This will not eliminate bias but it will minimize it.

It is even likely, albeit indemonstrable in the absence of a practical reference standard, that the more histopathologists know about a given MSN the higher the chance of a correct histopathologic diagnosis. In the present study, we have at least demonstrated that clinical information can improve the histopathologists' LDC, if not their own diagnosis. A LDC is currently not provided in histopathologic reports. Nonetheless, in routine practice any diagnostic decision is given by the balance between certainty and uncertainty. Some recently introduced histopathologic categories (superficial atypical melanocytic proliferation of uncertain significance, S.A.M.P.U.S.; melanocytic tumor of uncertain malignant potential, MEL.T.U.M.P.) [31] are basically the expression of a low LDC. Clinicopathologic correlation could probably raise the histopathologists' LDC in some of these highly controversial cases.

On average, in the stepwise examination of MSN each observer changed his initial diagnosis in about 7 cases. This means that in the course of the daily histopathologic examination of dozens of MSN, clinical information can probably induce to change the initial diagnosis in a substantial number of cases. In the present study, most diagnostic changes occurred in D2 (with the knowledge of the age and the sex of the patient and of the location of the lesion). The low number of diagnostic changes does not allow to draw any reliable conclusion, but we roughly assess that there are at least three types of melanocytic lesions which are particularly sensitive to clinical information, namely, lentiginous melanocytic proliferations [19], Spitz/Reed nevus-like neoplasms [4], [7], [18], and congenital nevi with atypical features.

As expected on the basis of the study selection criteria (i.e.: clinically and/or dermoscopically atypical MSN), the majority of the diagnostic switches in D4 (clinical image) and D5 (dermoscopic image) were to melanoma (see Table 2). Interestingly, some histopathologic diagnoses were switched into benignity after studying images from clinically/dermoscopically atypical lesions (see Table 2). We speculate that the very same submission of clinical/dermoscopic images to histopathologists can stimulate a critical re-evaluation of the histopathologic diagnosis just by introducing a new step of microscopic observation following the evaluation of clinical images.
