# Correction: The Influence of Clinical Information in the Histopathologic Diagnosis of Melanocytic Skin Neoplasms

**DOI:** 10.1371/annotation/512cb17b-934c-4a06-9dbb-114d43052a2b

**Published:** 2009-06-05

**Authors:** Gerardo Ferrara, Zsolt Argenyi, Giuseppe Argenziano, Rino Cerio, Lorenzo Cerroni, Arturo Di Blasi, Elisa A. A. Feudale, Caterina M. Giorgio, Cesare Massone, Oscar Nappi, Carlo Tomasini, Carmelo Urso, Iris Zalaudek, Harald Kittler, H. Peter Soyer

The author contributions are incorrect. The correct contributions are: Performed the experiments: ZA RC LC ADB EAAF ON CT CU HK HPS

